# Hydrocephalus and myelopathy: A rare and challenging case of sarcoidosis and review of literature

**DOI:** 10.1002/ccr3.3003

**Published:** 2020-06-05

**Authors:** Nasam Alfraji, Steven Douedi, Mohammad A. Hossain

**Affiliations:** ^1^ Department of Medicine Jersey Shore University Medical Center Hackensack Meridian Health Neptune NJ USA

**Keywords:** autoimmune, hydrocephalus, myelopathy, neurosarcoidosis, sarcoidosis

## Abstract

Neurological involvement is a rare presentation of sarcoidosis. Physicians should consider sarcoid as a cause of myelitis and hydrocephalus as early management with steroid improves patient survival and reduces long‐term disability.

## Graphical abstract

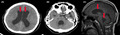

## 1 INTRODUCTION

Sarcoidosis is a multisystem autoimmune granulomatous disease. Primary neurologic involvement is a rare presentation. We present a challenging case of neurosarcoidosis presenting as acute hydrocephalus with a subsequent myelopathy. High suspicion is mandated in patients with primary myelitis and hydrocephalus as a prompt treatment with steroid would improve patient outcome.

Sarcoidosis is a rare multisystem autoimmune granulomatous disease with unknown etiology characterized by the presence of noncaseating granulomatous inflammation of involved organs.^1^ The respiratory system is affected in 95% of cases, with the skin, eye, lymph nodes, and liver next most commonly affected organs.^1^ The neurologic involvement occurs in 25% of patients with sarcoidosis on autopsy, but approximately 5%‐10% of all patients with sarcoidosis have neurologic symptoms.^1^, ^2^ The prevalence of sarcoidosis is three times more common in African Americans compared to Caucasians of Northern European descent.^1^ It commonly presents between 25 and 40 years old in both genders equally.^3^

Neurosarcoidosis (NS) can manifest most commonly as cranial neuropathies, especially unilateral facial nerve palsy.^4^ Other manifestations of NS can be optic neuropathy, peripheral neuropathy, meningeal involvement, encephalopathy, hydrocephalus, and myelopathy.^4^ Hydrocephalus and spinal cord involvement are the rarest presentations of NS.^3^, ^4^ Hydrocephalus, either communicating or noncommunicating, has been noted in 6%‐9% of cases of NS,^4^, ^5^ while spinal cord involvement occurs in approximately 6%‐8% of NS cases^6^ and occurring in <1% of all sarcoidosis cases.^3^

The clinical presentations of NS can be very broad and atypical, which makes the diagnosis of such disease exceedingly challenging especially in previously healthy individuals who come with hydrocephalus and/or myelopathy as initial manifestations without other evident symptoms of sarcoidosis.^4^, ^5^, ^7^

We report a rare case of NS with hydrocephalus and subsequent myelopathy as the initial presentations of the disease to feature the challenge of diagnosing NS in the absence of known systemic sarcoidosis and to emphasize the importance of timely diagnosis and appropriate treatment with steroid in reducing subsequent neurological sequelae.

## 2 CASE PRESENTATION

A 38 ‐year‐old an African American male with a past medical history of two years old motor vehicle accident with residual chronic low back pain, who presented to the emergency department with one‐day acute bilateral lower extremity weakness. The patient stated that he started feeling numb and heavy on the left lower extremity, which slowly progressed to both limbs and ascended upward. The patient reported one episode of bowel incontinence but denied any urinary incontinence.

The patient reported no fever, rash, recent trauma, weight/appetite change, and shortness of breath, cough, chest pain, nausea/vomiting, diarrhea, recent upper respiratory tract infection, and gastrointestinal or genitourinary infections. He did not report any redness of the eye, changes in vision, or dizziness. He denied any sick contacts, recent travel, or exposure to molds, chemicals, or asbestos. Family history was significant for stroke in mother and unknown type of cancer in father. He is married and works as a driver. He uses alcohol and cannabis occasionally but denied other recreational drugs use or tobacco use.

On examination, his vital signs were within normal limits. The patient was alert and oriented to time, place, and person. Head and neck examinations were unremarkable apart from a left cranial scar with a shunt underneath the skin. No lymphadenopathy was appreciated. Cardiology, pulmonary, and gastrointestinal examinations were unremarkable, and skin examination revealed no rashes. On neurological examination, his speech was fluent and coherent with intact memory. There was no neck stiffness or range of motion deficits. Cranial nerve examination was unremarkable. Strength was 5/5 in the bilateral upper extremities and 4/5 in bilateral lower extremities, with normal bulk and tone. No pronator drifts. Reflexes were graded 2 + throughout all extremities. Sensation for pinprick, temperature, and light touch was decreased in the left foot comparing to the right side. No gross dysmetria or ataxia.

**FIGURE 1:**
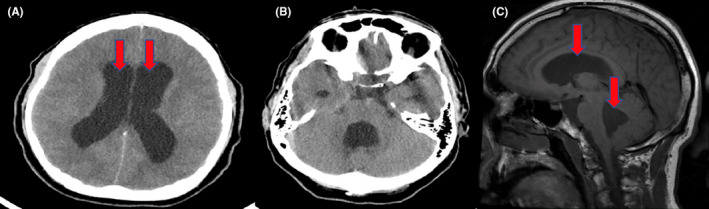
A and B, Dilation of fourth and lateral ventricles on CT head without contrast. C, Enlargement of fourth and lateral ventricles on T1 sagittal view of MRI head

Of note, the patient was admitted less than a year ago with a diagnosis of acute communicating hydrocephalus (Figure 1) when he presented with confusion and headache. He underwent a ventriculoperitoneal shunt insertion at that time. The patient had no focal neurological deficit during that admission. Patient was stable for discharge and to follow up outpatient. However, patient lost follow‐up afterward due to insurance/financial issues and definitive diagnosis was not made at that time.

During the 2nd hospital admission, patient developed episodes of urinary retention requiring a Foley catheter to be placed. Computerized tomography (CT) head without contrast showed no evidence of acute territorial infarction or bleed. The fourth and lateral ventricles were stable in size. There was no midline shift or mass effect. Complete shunt series showed intact right ventriculoperitoneal shunt. MRI of the head with and without contrast was compatible with CT head report.

**FIGURE 2:**
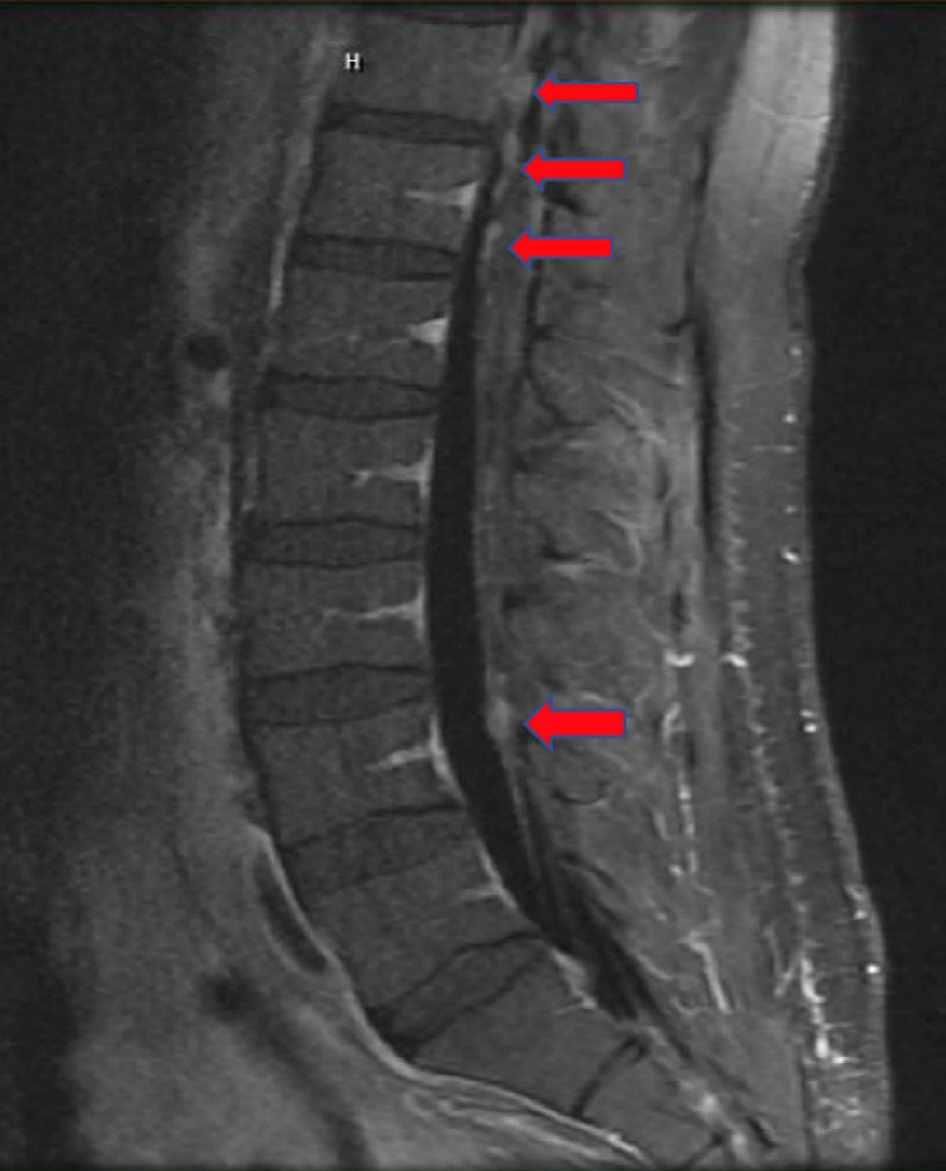
Sagittal T1 Flair view of MRI lumbar with contrast showing small enhancing nodules from T11 through L4 suspicious for leptomeningeal drop metastases. No compressive lesion seen

An MRI of the cervical/thoracic/lumbar spine with and without contrast showed an enhanced nodular curvilinear area in the inferior fourth ventricle (Figure 2). Small 2‐ to 3‐mm enhancing nodules were seen along the surface of the posterior cord at C3‐C4 and around the cord from C6 through T1‐T2 level as well as from T1 to L4 suspicious for leptomeningeal involvement. Tiny enhancing nodules were most concentrated in the distal thoracic spine to upper cauda equina. No paravertebral masses or compressive lesions were seen.

Lumbar puncture showed a clear CSF, elevated white blood cell (WBC) count at 83 with predominantly 97% lymphocytes, slight xanthochromia, glucose of 25, and a CSF total protein elevated at 298 (Table 1). CSF cultures and cytometry were negative. HIV serology and Quantiferon gold testing were normal. His complete blood count (CBC) and chemistry including RFT and LFT were within normal limits. Syphilis and Lyme titers were nonsignificant. angiotensin‐converting enzyme (ACE) at 40 U/L (9‐67 U/L), anti‐nuclear antibodies (ANA) 0.23 (0.0‐0.90), C‐reactive protein (CRP) of 0.23 mg/dL (0‐0.744 mg/dL), and erythrocyte sedimentation rate (ESR) of 3 mm/h (0‐15 mm/h). Vitamin B12, folate, and TSH were within normal ranges.

**TABLE 1:** Summary of CSF analysis

Laboratory value	Results	Reference value
CSF appearance	Clear	‐
CSF RBC	Occasional/few (45‐150/μL)	0/μL
CSF WBC	83	0‐5/mm^3^
CSF glucose	25	50‐75 mg/dL
CSF total protein	298	14‐45 mg/dL
CSF segs	0	0%‐6%
CSF lymphocytes	97	48%‐72%
CSF eosinophils	0	‐
CSF bands	0	‐
CSF monocytes	3	26%‐35%
CSF macrophages	0	‐
CSF blast cells	0	‐
CSF albumin	169	0‐35 mg/dL
CSF IgG	52.3	0‐6 mg/dL
CSF IgG/albumin ratio	0.31	0.09‐0.25

**FIGURE 3:**
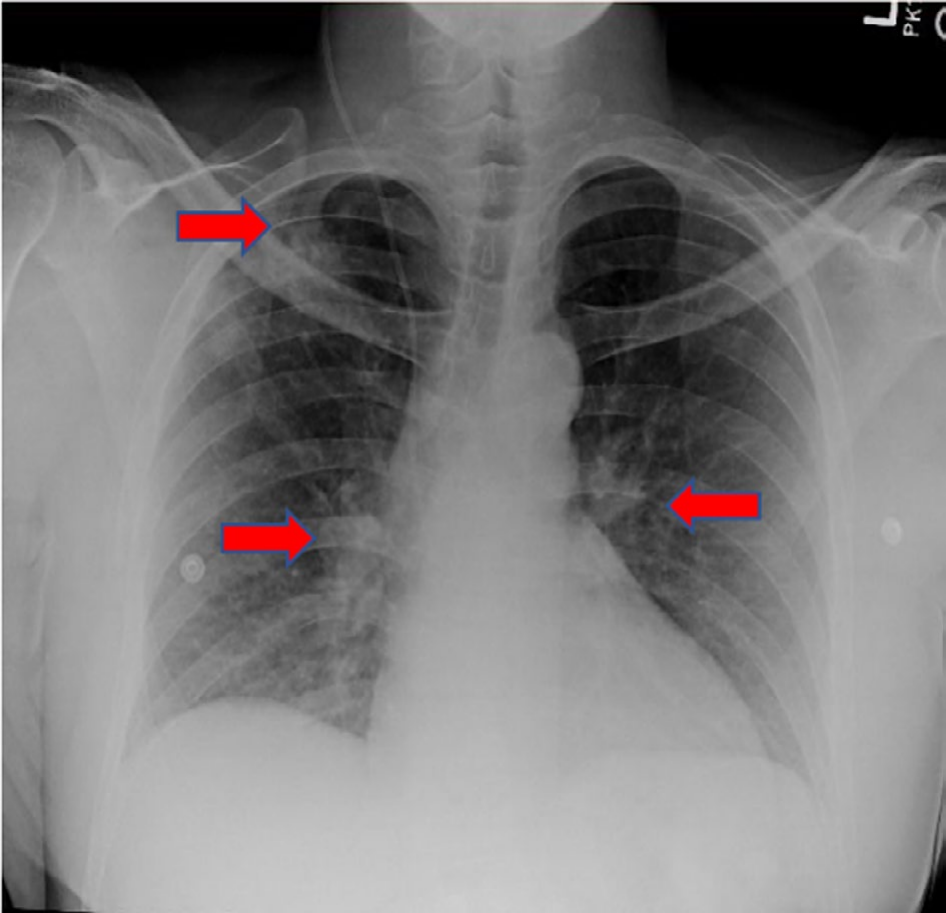
Chest X‐ray revealing bilateral fullness of the pulmonary hila with patchy opacity within the right upper lobe

**FIGURE 4:**
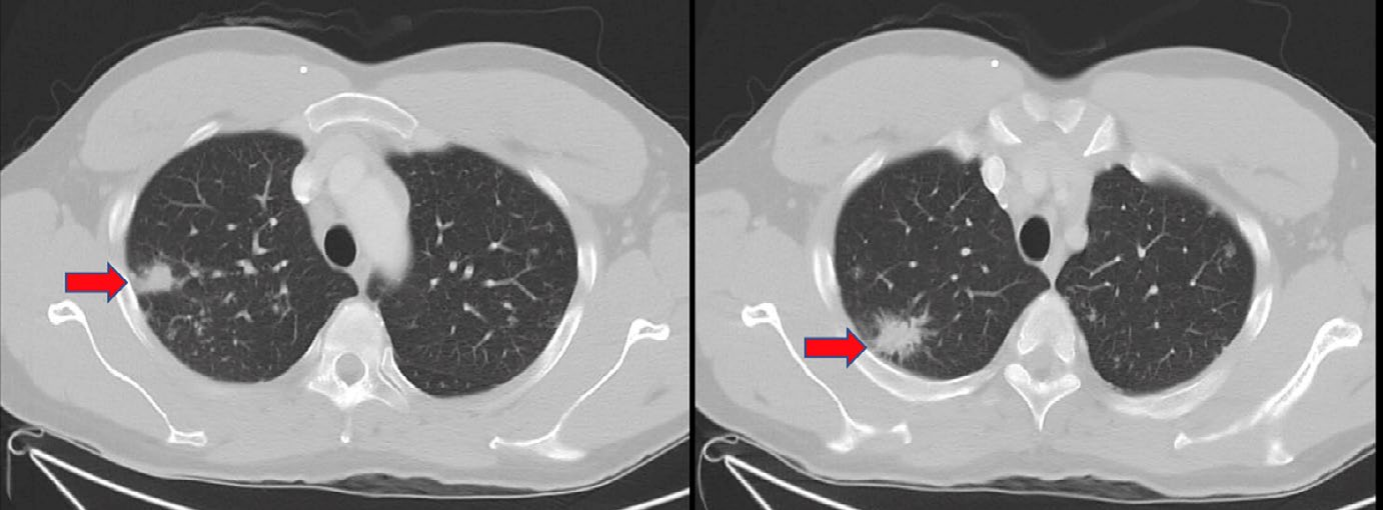
CT chest/abdomen/pelvis with contrast revealing enlarged a 1.8‐cm speculated nodule within the right upper lobe, with a 1.7‐cm nodule more inferiorly within the right upper lobe

Chest X‐ray (Figure 3) showed bilateral fullness of the pulmonary hila and patchy opacity within the right upper lobe. CT chest with contrast showed enlarged mediastinal and hilar lymph nodes, with incidental multiple nodular densities within the lungs predominantly within the right upper lobe (Figure 4). Endobronchial ultrasound‐guided fine‐needle aspiration (FNA) biopsy of the right paratracheal and hilar lymph nodes was obtained. The biopsy was significant for reactive bronchial cells admixed with multinucleated giant cells and rare epithelioid macrophages on a background of lymphocytes suggestive of noncaseating granulomatous inflammation. Flow cytometry was normal. Acid‐fast bacilli (AFB) stain and bronchoalveolar lavage cultures were negative.

During his hospital stay, he had progressive worsening in sensation up to the waist with worsening weakness of his lower extremities as his strength at lower extremities became 0/5, but he preserved his full strength at upper extremities bilaterally. The neurogenic bladder was persistent, requiring indwelling Foley catheter. Patient also developed constipation and was placed on several bowel regimens.

Therefore, in view of the patient's history, the imaging, laboratory investigations, CSF analysis, and lymph node biopsy result, neurosarcoidosis was probable diagnosis according to the modified Zajicek criteria.^8^ However, a neoplastic process was still in the differential considerations. Thus, spinal cord biopsy was recommended to confirm the diagnosis of neurosarcoidosis, but it was declined by the patient. The case was discussed among different specialties including the medicine, the rheumatology, hem/oncology, neurology, and neurosurgery departments, and consensus was made to start high‐dose pulse intravenous steroid and to transfer the patient to a tertiary medical center for a higher level of care.

He was treated with a course of steroid and discharged to rehab facility on a slow oral prednisone taper. At his latest follow‐up, several months later, he reported a partial improvement in his sensory symptoms, but there was not much improvement in lower extremity motor weakness. However, there was no worsening of his overall neurological picture.

## 3 DISCUSSION

Sarcoidosis is a multiorgan inflammatory disease that is commonly diagnosed in young and middle‐aged adults, especially in the African American population. Diagnosis of sarcoidosis is achieved after fulfilling the diagnostic criteria of positive radiologic findings, pathology showing noncaseating granulomas, and exclusion of other differentials such as infections and malignancies.^9^ Neurologic manifestations due to systemic sarcoidosis are far more rare and lethal finding, occurring in 5%‐15% of systemic sarcoidosis cases.^10^, ^11^ In addition to systemic symptoms, a systematic review by Mubarik et al reported neurosarcoidosis (NS) commonly presenting as a cranial nerve palsy, usually the facial nerve in up to 50% of patients, headache, and sensory or motor impairment in about 30% of cases.^12^ NS presenting with neurological symptoms alone and with hydrocephalus and spinal cord involvement, as seen in our patient presented, is far more uncommon occurring in only about 1% of cases.^4^, ^13^ The diagnosis of NS has been made by using the modified Zajicek criteria.^8^ This criterion has been categorized by “definitive,” “probable,” and “possible.” Definitive diagnosis has been defined as being made with a positive neural biopsy and histopathology after excluding other causes and differential diagnoses. Probable diagnosis defined as positive imaging studies, such as MRI, high‐resolution CT scan, or FDG‐PET scan, and positive extra‐neural biopsy with exclusion of differentials. A possible diagnosis relies on clinical presentation and exclusion of differentials.^8^, ^14^

Corticosteroids are suggested as the first‐line treatment for NS, usually at a dose of 1 mg/kg/dose of prednisolone with a taper for 6‐8 months.^10^ However, long‐term use of steroids has been associated with severe and debilitating side effects, and therefore, second‐line treatment with methotrexate, cyclophosphamide, and other immunosuppressive agents has been used.^15^ TNF‐alpha antagonists are beginning to be used in randomized clinical control trials in combination with first‐ and second‐line therapies; however, there is still no well‐structured guidelines in mainstreaming their use.^11^

Early treatment with first‐line therapy has been shown to lead a substantial improvement of symptoms and survival in patients diagnosed with NS. In a meta‐analysis by Fritz et al, 465 treatments for patients with neurosarcoidosis were examined and 27% of patients went into disease remission after early treatment. Out of 227 patients receiving only first‐line treatment, 71% had favorable outcomes.^11^ Despite these positive outcomes, there has yet to be a defined standard for the treatment of neurosarcoidosis.^13^

The patient presented in our unique case report only had neurological symptoms on presentation.

He had a positive extra‐neural biopsy and imaging studies including MRI that lead to a “probable” diagnosis of neurosarcoidosis as a neural biopsy was declined by the patient. Diagnosis of NS by imaging and extra‐neural biopsy using the modified Zajicek criteria and exclusion of other differential diagnoses led to a rapid identification and treatment with first‐line therapy using glucocorticoids. However, the patient had minimal improvement in his neurological symptoms and required rehab for further management while on a steroid taper.

On review of literature, there are few cases of NS presented with either myelopathy or hydrocephalus before a diagnosis of sarcoidosis was made, but no case reported with both myelopathy and hydrocephalus as isolated presentations of NS and without a previous diagnosis of sarcoidosis.^4^, ^5^, ^6^, ^8^, ^14^ Our case was similar to the other reported cases regarding the presenting manifestations of weakness and/or sensory symptoms of extremities in the setting of myelopathy as well as headache and/or confusion in the setting of hydrocephalus in the absence of evident systemic disease. However, the response to treatment in each case was variable. According to the same abovementioned meta‐analysis by Fritz et al, one of the recent studies discussing NS outcomes, complete/partial remission was noted in 59%, stability in 24%, and progressive deterioration in 6% of treated individuals.^11^

Despite advancements in medicine and neurosarcoidosis studies, accurate diagnostic criteria and standardized treatment regimens have been lacking due to insufficient clinical trials.

## 4 CONCLUSION

Given the variability of NS symptomatology and the unavailability of standardized diagnostic criteria, physicians must include sarcoidosis highly in their differential considerations after excluding common infectious, neoplastic, and other autoimmune disorders, especially for African American patients, aged 25‐40 years, who present with unexplained neurological symptoms. Although we have insufficient randomized controlled trials guiding NS treatment, early diagnosis, timely treatment with steroids, and a referral to a specialist center can be associated with a favorable outcome and prevent further deterioration and long‐term disability. This case necessitates further case studies and research in this topic.

## CONFLICT OF INTEREST

The authors declare that there is no conflict of interests regarding the publication of this paper.

## AUTHOR CONTRIBUTION

NA and SD: were involved in the case selection, in addition to writing and reviewing the manuscript. MAH: worked on the planning, manuscript revision, and final approval. All authors were involved in the editing and final approval of the manuscript.

## ETHICAL APPROVAL

The patient described in the case report had given informed consent for the case report to be published.
